# Metabolite Responses to Exogenous Application of Nitrogen, Cytokinin, and Ethylene Inhibitors in Relation to Heat-Induced Senescence in Creeping Bentgrass

**DOI:** 10.1371/journal.pone.0123744

**Published:** 2015-03-30

**Authors:** David Jespersen, Jingjin Yu, Bingru Huang

**Affiliations:** 1 Department of Plant Biology and Pathology, Rutgers University, New Brunswick, NJ, United States of America; 2 College of Agro-Grassland Science, Nanjing Agricultural University, Nanjing, P.R. China; Chinese Academy of Sciences, CHINA

## Abstract

The exogenous application of ethylene inhibitors, cyotkinins, or nitrogen has previously been shown to suppress heat-induced senescence and improve heat tolerance in cool -season grasses. The objectives of this study were to examine metabolic profiles altered by exogenous treatment of creeping bentgrass with an ethylene inhibitor, cytokinin or nitrogen under heat stress and to determine metabolic pathways regulated by those compounds in association with their effectiveness for improving heat tolerance. Creeping bentgrass (*Agostis stolonifera*) plants (cv. Penncross) were foliar sprayed with 18 mM carbonyldiamide (N source), 25μM aminoethoxyvinylglycine (AVG, ethylene inhibitor), 25μM zeatin riboside (ZR, cytokinin), or a water control, and then exposed to 20/15°C (day/night) or 35/30°C (heat stress) in growth chambers. All three exogenous treatments suppressed leaf senescence, as manifested by increased turf quality and chlorophyll content, and reduced electrolyte leakage under heat stress. Polar metabolite profiling identified increases in the content of certain organic acids (i.e. citric and malic acid), sugar alcohols, disaccharides (sucrose), and decreased accumulations of monosaccharides (i.e. glucose and fructose) with exogenous treatment of N, AVG, or ZR at the previously mentioned concentrations when compared to the untreated control under heat stress. Nitrogen stimulated amino acid accumulation whereas AVG and ZR reduced amino acid accumulation compared to the untreated control under heat stress. These results revealed that the alleviation of heat-induced leaf senescence by N, AVG, and ZR could be due to changes in the accumulation of metabolites involved in osmoregulation, antioxidant metabolism, carbon and nitrogen metabolism, as well as stress signaling molecules.

## Introduction

Heat is a major abiotic stress which leads to premature leaf senescence in many plant species, including cool-season grass species. Stress-induced senescence is associated with metabolic changes and shifts in carbon relations, the production of damaging reactive oxygen species, as well as the degradation of cellular constituents including proteins, lipids, and pigment molecules such as chlorophyll [1]. Many approaches have been used to alleviate heat stress-induced senescence and improve whole-plant stress tolerance, including exogenous application of plant growth regulators or hormones, such as ethylene inhibitors and cytokinins and nutrients, such as nitrogen [2–4]. However, metabolic mechanisms associated with heat-induced leaf senescence that are regulated by those external compounds are not well understood.

Ethylene is a gaseous plant hormone affecting plant stress tolerance and regulating senescence [5, 6]. An increased level of ethylene is one of the main signals for inducing senescence [7]. Ethylene levels have also been shown to increase in response to a number of abiotic stresses, including heat, drought, and salinity [8, 9]. Decreased levels of ethylene production have been associated with improved stress tolerance in a cool-season grass species [10]. Aminoethoxy vinyl glycine (AVG) is an ethylene inhibitor which reduces ethylene levels by inhibiting ethylene biosynthesis [11]. The use of AVG to reduce endogenous ethylene levels delay senescence has been demonstrated in a number of species including oat (*Avena sativa)*, wheat (*Triticum aestivum*), and creeping bentgrass [2, 12, 13]. However, specific metabolites regulated by an ethylene inhibitor that may suppress heat-induced leaf senescence are yet to be determined.

Cytokinins are another class of plant hormones which play important roles in cell differentiation and division [14]. Cytokinins also play a role in stress response and delaying leaf senescence [15]. Both exogenous applications of cytokinins, as well as genetic transformation to increase endogenous cytokinins levels have been shown to delay senescence and increase abiotic stress tolerance in creeping bentgrass [2, 16–18]. It is unclear how exogenous application of cytokinins may control leaf senescence induced by heat stress at the metabolic level.

Nitrogen is a major growth limiting nutrient for plants which is an important constituent of biomolecules such as nucleic acids and proteins [19]. Nitrogen is well known for its effects on reducing leaf senescence during heat stress conditions [20]. Nitrogen has been shown to improve heat tolerance by increasing antioxidant and photosynthetic activities in perennial grass species [18, 21], and resulting in elevated accumulations of heat shock proteins in *Zea mays* [22]. However, metabolic responses to additional nitrogen related to leaf senescence under heat stress are not well documented.

We have previously reported alteration of proteomic profiles by the exogenous application of ethylene inhibitors, cytokinins, or nitrogen in relation to the suppression of heat-induced leaf senescence in a cool-season grass species, creeping bentgrass (*Agrostis stolonifera*) [4]. The previous study reported that the alleviation of heat-induced senescence by N, AVG, or ZR was associated with enhanced protein abundance in photosynthesis and amino acid metabolism and stress defense systems (heat shock protection and antioxidants), as well as suppression of those imparting respiration metabolism. However, specific metabolites responsive to N, AVG, or ZR involved in those important metabolic pathways, such as photosynthesis and respiration, identified through the proteomic analysis are unknown. Metabolomic profiling is a powerful approach for identifying metabolites and metabolic pathways regulating plant growth and responses to external stimuli or stresses [23]. Creeping bentgrass is a widely used forage and turf grass species in temperate regions, but sensitive to high temperatures, and understanding mechanisms of improving heat tolerance is important for enhancing the productivity and quality of cool-season grass species in environments with increasing temperatures. The objective of this study was to identify metabolites and associated metabolic pathways affected by the exogenous application of an ethylene inhibitor, cytokinin, nitrogen compounds that may contribute to their effects on the suppression of heat-induced leaf senescence and plant tolerance to heat stress in a cool-season perennial grass species, creeping bentgrass (*Agrostis stolonifera*).

## Materials and Methods

### Plant materials and growth conditions

Sod plugs of creeping bentgrass (cv. ‘Penncross’) were collected from mature field plots at the Rutgers University Hort Farm II research facility, North Brunswick, NJ. Plants were transplants in to plastic pots filled with fine sand (15 cm in diameter and 20 cm deep) and allowed to establish in a greenhouse. During the 30-d establishment, plants were watered three times each week, received Hoagland’s nutrient solution weekly [24], and were trimmed to maintain a 5-cm canopy height. Plants were then transferred to controlled environment growth chambers (Conviron, Winnipeg, Canada) set at 20/15°C (day/night temperature), a 14-h photoperiod with 610 μmol m^-2^ s^-1^ photosynthetically active radiation (PAR) and allowed to acclimate for one week before the beginning of treatments.

### Treatments and Experimental design

Plants were treated with an ethylene inhibitor, aminoethoxyvinyl glycine (AVG), at 25 μM, cytokinin (trans-zeatin riboside, ZR) at 25 μM, nitrogen (N) (carbonyldiamide, urea) at 18 mM, and water (untreated control) daily for 3 d prior to heat stress treatments, and then were applied at a 7-d interval for the remainder of the 28-d of heat treatment. AVG and N were prepared in water, and ZR was dissolved in trace amount of 1N NaOH, before being diluted to appropriate concentrations with water. The ZR and AVG concentrations were selected based on preliminary tests showing positive effects on suppressing leaf senescence under heat stress [2], [4]. The N rate was selected based on the common recommendation of N rate for foliar application in creeping bentgrass used as golf turf. Chemicals were obtained from Sigma-Aldrich (St. Louis, MO). Additionally, all treatments contained 0.05% Tween 20. All treatments were applied as foliar spray at a volume which saturated the canopy (approximately 375 ml m^-2^).

Following 3-d treatment with water, AVG, N, or ZR, plants were exposed to two temperature treatments for 28 d: 20/15°C (day/night) as the temperature control, or 35/30°C (heat stress). Other growth chamber conditions were the same as described above. During the treatment period plants were watered daily, and fertilized twice per week with ¼ strength Hoagland’s nutrient solution to maintain adequate hydration and nutrient status.

The experimental design was a split-plot design, with temperature treatments as the main plots, and exogenous treatments as the sub-plots. Each temperature treatment was repeated in four growth chambers. Each exogenous treatment had four replicates (four pots with multiple plants in each pot) within each temperature treatment. Plants from each temperature treatment were relocated among four growth chambers every 5 d to avoid potential confounding effects of environmental variations among different growth chambers.

### Physiological measurements

Leaf senescence and whole-plant heat tolerance was evaluated using three commonly-used indicators, including visual ratings of turf quality, chlorophyll content and cell membrane stability. Measurements were taken every 7-d starting at the onset of temperature treatments to assess treatment effects. Turf grass quality (TQ) was visually rated as an indicator of overall plant vigor based on color, density and uniformity of grass canopy on a 1–9 scale, with 9 being the best in all three quality components and 1 being dead plants [25].

Chlorophyll content was measured using a modification of the methods described by [26]. 0.1 gram of fresh leaf tissue was incubated in 10 ml of dimethyl-sulfoxide in the dark for 72 hours to extract chlorophyll from the leaf tissue. The resulting solution was measure on a spectrophotometer (Spectronic Instruments, Inc., Rochester, NY) at 663nm and 645nm. Leaf tissue was then dried for 72 h in a 80°C oven to obtain dry weights. Chlorophyll content was calculated on a fresh weight basis using the equations described by [27].

Membrane stability was estimated as electrolyte leakage using the methods described by [28]. About 0.1g of fresh leaf tissue was placed in a tube with 35 ml de-ionized water and placed on an orbital shaker for 16h. An initial conductance reading (C_initial_) of the incubated solution was taking using a conductivity meter (YSI Incorporated, Yellow Springs, OH). Tubes were then autoclaved at 120°C for 20 min to kill all contained leaf tissue. Tubes were places back on the shaker for an additional 16 h and a final conductance reading was measured (C_max_). Electrolyte leakage was calculated as a percentage of C_initial_ to C_max._ to determine the percent of relative damage.

### Metabolite analysis

#### Extraction and derivatization of metabolites

Metabolites associated with heat-induced leaf senescence that may be regulated by nitrogen, cytokinin, and the ethylene inhibitor were identified using gas chromatography and mass spectrometry analysis. The procedure was conducted following the method used by [29]. Leaf tissue samples from 28 d of heat treatment were harvested and immediately frozen in liquid nitrogen and stored at -80°C for later metabolite analysis. The extraction was modified from previously described protocols [30, 31]. For each sample, frozen leaves were lyophilized using a FreeZone 4.5 system (Labconco, Kansas City, MO) then ground to a fine powder, 25 mg of leaf tissue powder was transferred into a 10 ml microcentrifuge tube, and extracted in 1.4 ml of 80% (v/v) aqueous methanol at ambient temperature under 200 rpm for 2 h. Ribitol (10 μl of a 2 mg ml^-1^ solution) was added to each sample as an internal standard prior to incubation. Then samples were extracted in a water bath for 15 min at 70°C. Tubes were centrifuged for 30 min at 12,000 rpm, the supernatant was transfered into new tubes, and 1.4 ml of water and 0.75 ml of chloroform were added. The mixture was thoroughly vortexed and centrifuged for 5 min at 5,000 rpm. 2 ml of the polar phase (methanol/water) was decanted into 1.5 ml HPLC vials and dried in a Centrivap benchtop centrifugal concentrator (Labconco, Kansas City, MO). The dried polar phase was methoximated with 80 μl of methoxyamine hydrochloride (20 mg ml^-1^) at 30°C for 90 min and was trimethylsilylated with 80 μl N-Methyl-N-trimethylsilyltri-fluoroacetamide (with 1% trimethylchlorosilane) for 60 min at 70°C.

#### Gas chromatography mass spectrometry (GC-MS) analysis

GC-MS analysis followed the procedure described in [32]. The derivatized extracts were analyzed with a PerkinElmer gas chromatograph coupled with a TurboMass-Autosystem XL mass spectrometer (PerkinElmer Inc., USA). Extract aliquots of 1 ul were injected into a DB-5MS capillary column (30 m × 0.25 mm × 0.25 μm, Agilent J & W Scientific, Folsom, CA). The inlet temperature was set at 260°C. After a 6.5 min solvent delay, initial GC oven temperature was set at 60°C; 1 min after injection, the GC oven temperature was raised 5°C min^-1^, and finally held at 280°C for 15 min. The injection temperature was set to 280°C and the ion source temperature was adjusted to 200°C. The helium carrier gas had a constant flow rate of at 1ml min-1. The measurements were made with electron impact ionization (70 eV) in the full scan mode (m/z 30–550). Turbomass 4.1.1 software (PerkinElmer Inc., USA) coupled with commercially available compound libraries: NIST 2005, Wiley 7.0 was used to identify the detected metabolites. For GC/MS results, compounds were identified based on retention times and comparison with reference spectra in mass spectral libraries. Peaks areas of metabolites were integrated with the Genesis algorithm, and relative quantities were calculated using the ribitol internal standard.

### Statistical analysis

Treatment effects were analyzed using an ANOVA test and treatments found to have significant difference were tested by Fisher’s protected LSD at α = 0.05 to separate the means. Principle component analysis (PCA) using a correlation matrix to standardize the variables was performed to identify components which contributed the greatest variance between treatment groups. Additionally partial least squares regression (PLS) was used to model the relationship between metabolites and physiological measurements for overall heat tolerance. Variable importance project (VIP) scores were calculated for the PLS model to determine which metabolites according to Wold’s criterion had higher contributions to delaying leaf senescence as estimated by chlorophyll content [33]. This approach accounts for both predictors and response variables to determine metabolites contribution to the model. Statistical analysis was performed using SAS v9.2 (SAS Institute Inc, Cary, NC).

## Results

### Effects of exogenous treatments on suppressing heat induced leaf senescence

**Fig 1:**
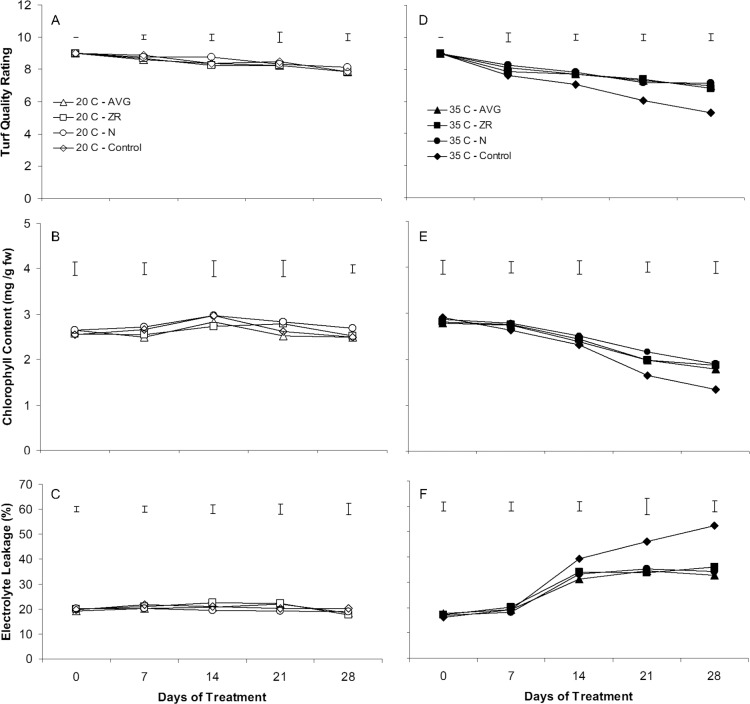
Physiological effects of AVG, CK, and N in creeping bentgrass. Changes to turf quality (A), chlorophyll content (B), and electrolyte leakage (C) during 28 d of 20/15 C or 35/30 C when treated with AVG, ZR, N or water as a control. Vertical bars represent least significance difference values between exogenous treatments at p = 0.05.

All growth and physiological measurements, TQ, chlorophyll content, and EL, remained relatively unchanged during the experimental period (28 d) under non-stress temperature (20/15°C) (Fig 1A, 1B and 1C). At this temperature, no significant differences in TQ, chlorophyll content or EL were detected between treatments of N, ZR, or AVG and the untreated water control (Fig 1A, 1B and 1C).

Turf quality declined in all treatments during heat stress but the decline happened sooner and to a greater degree in the untreated plants (Fig 1D). By 28 d of heat stress, TQ rating had dropped to 5.3 for untreated plants, but remained significantly higher in the N, ZR, and AVG treatments than the untreated control, with TQ being 7.1, 6.8, and 7.0, respectively.

Leaf chlorophyll content also declined in all treatments during heat stress (Fig 1E). By 28 d of heat stress, chlorophyll content declined by 59% in the untreated plants, whereas that for N, ZR, and AVG treatments declined by 41, 41, and 43% respectively. Plants treated with N, ZR, or AVG exhibited significantly greater chlorophyll content than the untreated plants at 21 and 28 d of heat stress.

Electrolyte leakage (EL) increased in all treatments during heat stress, indicating a loss of membrane stability (Fig 1F). By 28 d of heat stress, EL increased to 33.0, 36.0, and 34.0% in AVG, ZR, and N treatments, respectively, while it increased to 52.4% in the untreated plants. Plants treated with N, ZR, or AVG had significantly lower EL at 21 and 28 d of heat stress compared to the untreated control.

### Metabolite responses to exogenous N, ZR, or AVG application under heat stress

Results of metabolic profiling are presented here for plants exposed to heat stress conditions, but not for leaves exposed to the non-stress temperature due to the lack of significant physiological effects of N, ZR, and AVG applications compared to control for non-stress treatments. The differential responses of metabolites to N, ZR, and AVG application during heat stress may highlight the metabolic processes or pathways associated with the positive physiological effects of N, ZR, and AVG application in suppressing leaf senescence and improving whole-plant heat tolerance, as discussed below.

**Fig 2:**
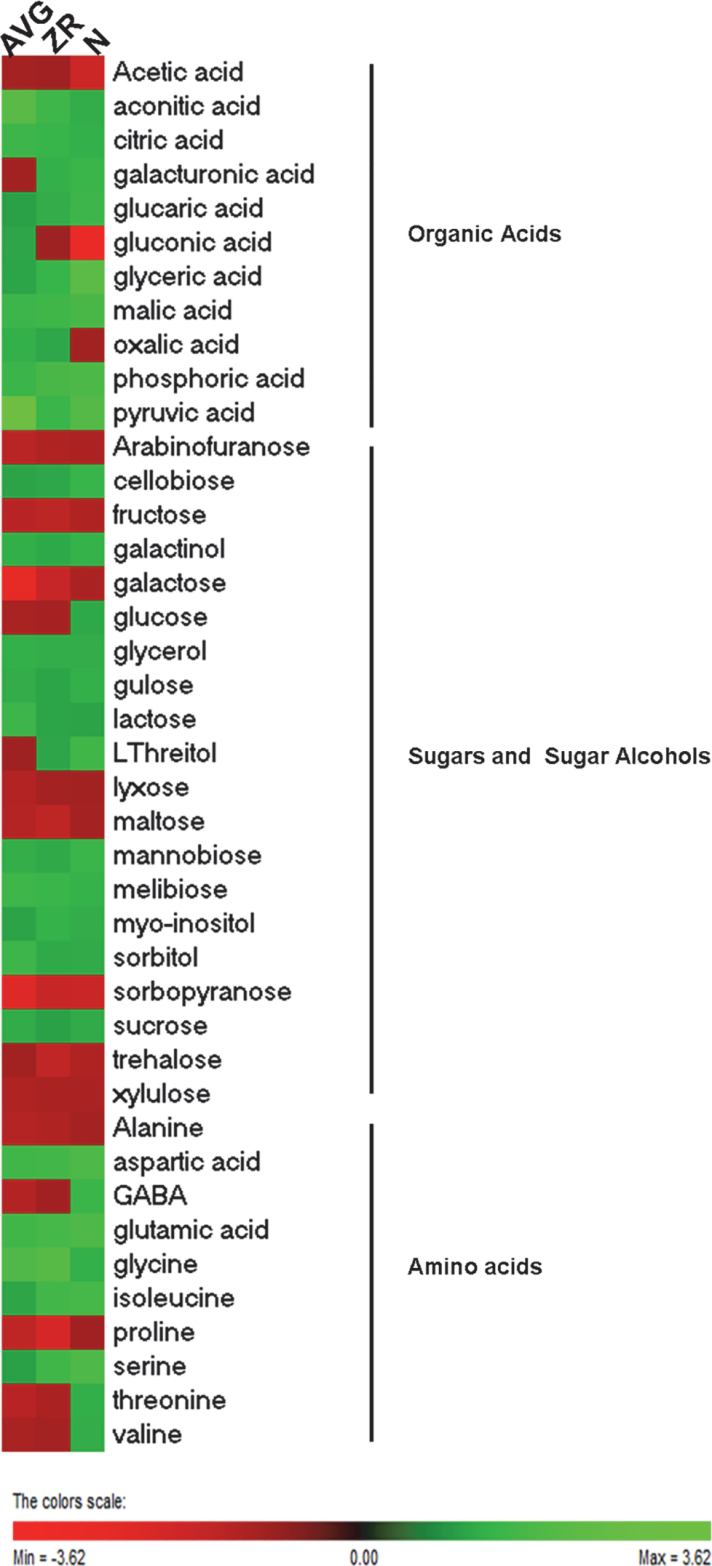
Heat map of changes in metabolite levels in AVG, ZR, and N-treated plants at 28-d heat stress compared to the control. Heat map showing the fold change of each metabolite for AVG, ZR, and N-treated plants when compared against the control at 28-d Heat stress. Green indicates an increase in metabolite fold number or up-regulation, and red indicates a down-regulation of a specific metabolite compared to the control.

A total of 41 polar metabolites were quantified in leaves of creeping bentgrass exposed to heat stress and treated with N, ZR or AVG, out of which 11 were organic acids, 20 were sugars or sugar alcohols, and 10 were amino acids (Table 1). Out of the 41 metabolites, 38 had significantly altered levels in N, ZR, or AVG treated plants compared to untreated plants (Fig 2, S1 Table). The application of N resulted in an increase in the content of 24 metabolites, a decreased content for 8 metabolites, and no significant effects on 9 metabolites relative to the untreated control at 28 d of heat stress (Fig 2). The application of ZR caused increases in the content of 15 metabolites, decreased content for 12 metabolites, and no significant effects for 14 metabolites (Fig 2). The application of AVG led to increases in the content of 15 metabolites and decreases in the content of 13 metabolites and did not have significant effects on 13 metabolites (Fig 2).

**Table 1:** The 41 metabolites identified by GC-MS at 28 d heat stress and there respective retention times (RT).

	**Metabolite**	**RT**
1	Acetic acid	9.33
2	Aconitic acid	26.36
3	Citric acid	27.84
4	Galacturonic acid	46.26
5	Glucaric acid	27.78
6	Gluconic acid	31.66
7	Glyceric Acid	16.28
8	Malic acid	20.37
9	Oxalic acid	11.06
10	Phosphoric acid	12.07
11	Pyruvic acid	8.62
12	Arabinofuranose	26.61
13	Cellobiose	43.45
14	Fructose	28.91
15	Galactinol	46.99
16	Galactose	29.27
17	Glucose	29.41
18	Glycerol	14.79
19	Gulose	34.87
20	Lactose	34.36
21	L-Threitol	26.15
22	Lyxose	24.79
23	Maltose	40.17
24	Mannobiose	42.54
25	Melibiose	48.91
26	Myo-inositol	33.09
27	Sorbitol	30.05
28	Sorbopyranose	27.71
29	Sucrose	42.22
30	Trehalose	43.69
31	Xylulose	22.44
32	Alanine	10.05
33	Aspartic acid	21.13
34	GABA	21.32
35	Glutamic acid	23.50
36	Glycine	10.57
37	Isoleucine	15.28
38	Proline	15.35
39	Serine	17.08
40	Threonine	17.75
41	Valine	13.14

**Fig 3:**
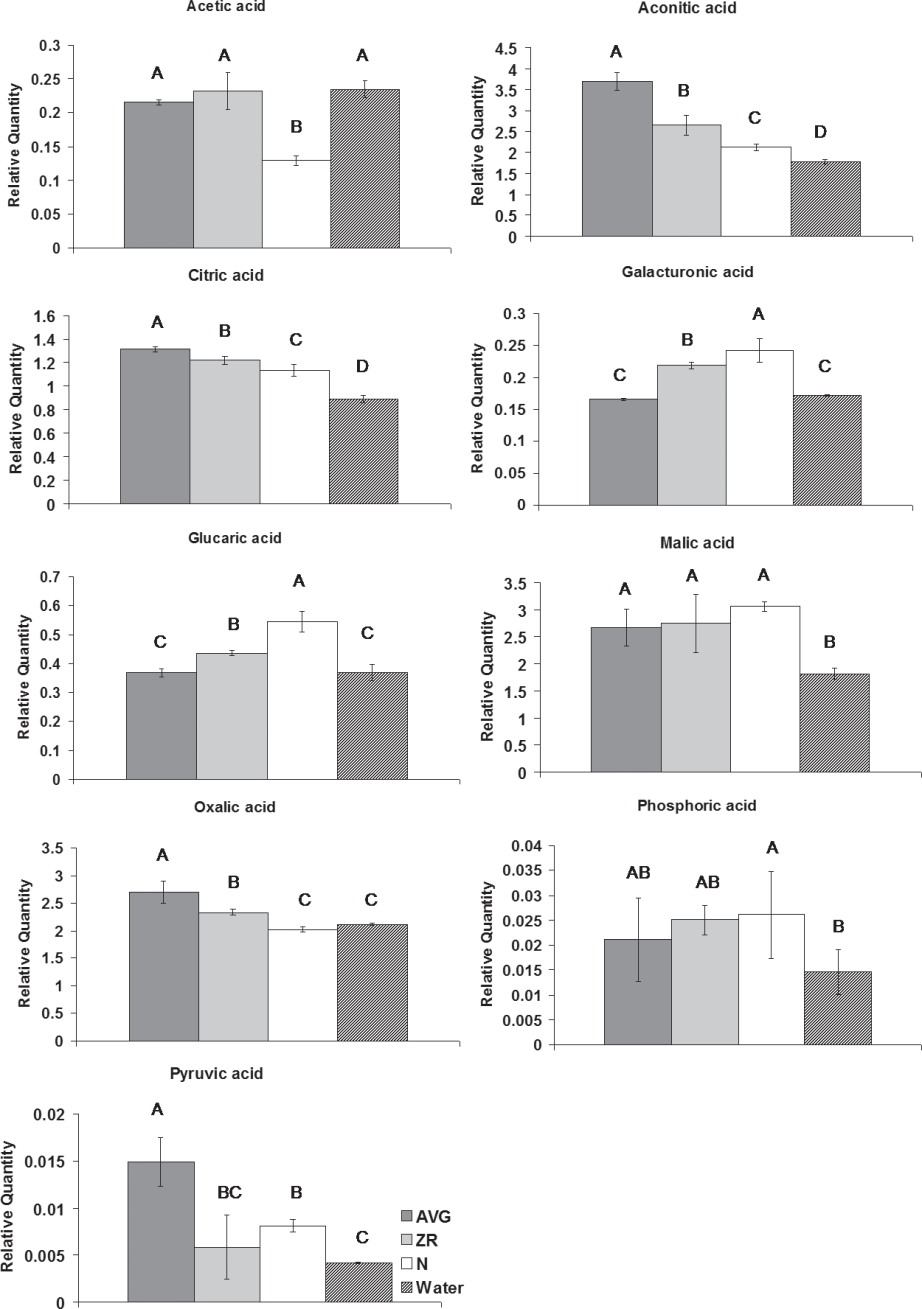
Organic acid levels during heat stress for AVG, ZR, and N-treated plants, and the water-treated control. Relative quantities of organic acids at 28-d heat stress for AVG, ZR, and N-treated plants, and the water-treated control. Error bars indicate standard deviations and letters are statistical groups according to Fisher’s protected LSD (p = 0.05), with groups not containing the same letter being significantly different. Only metabolites which had at least one group significantly different from the others are presented.

The application of N, AVG, or ZR resulted in significant increases in the content of a number of organic acids at 28 d of heat stress. All three exogenous treatments resulted in increased levels of aconitic acid, citric acid, and malic acid (Fig 3). Both AVG and ZR treatments caused an increase in oxalic acid, while AVG or N treatment resulted in an increase in pyruvic acid. Additionally, treatment with ZR or N lead to significant increases in galacturonic acid and glucaric acid.

**Fig 4:**
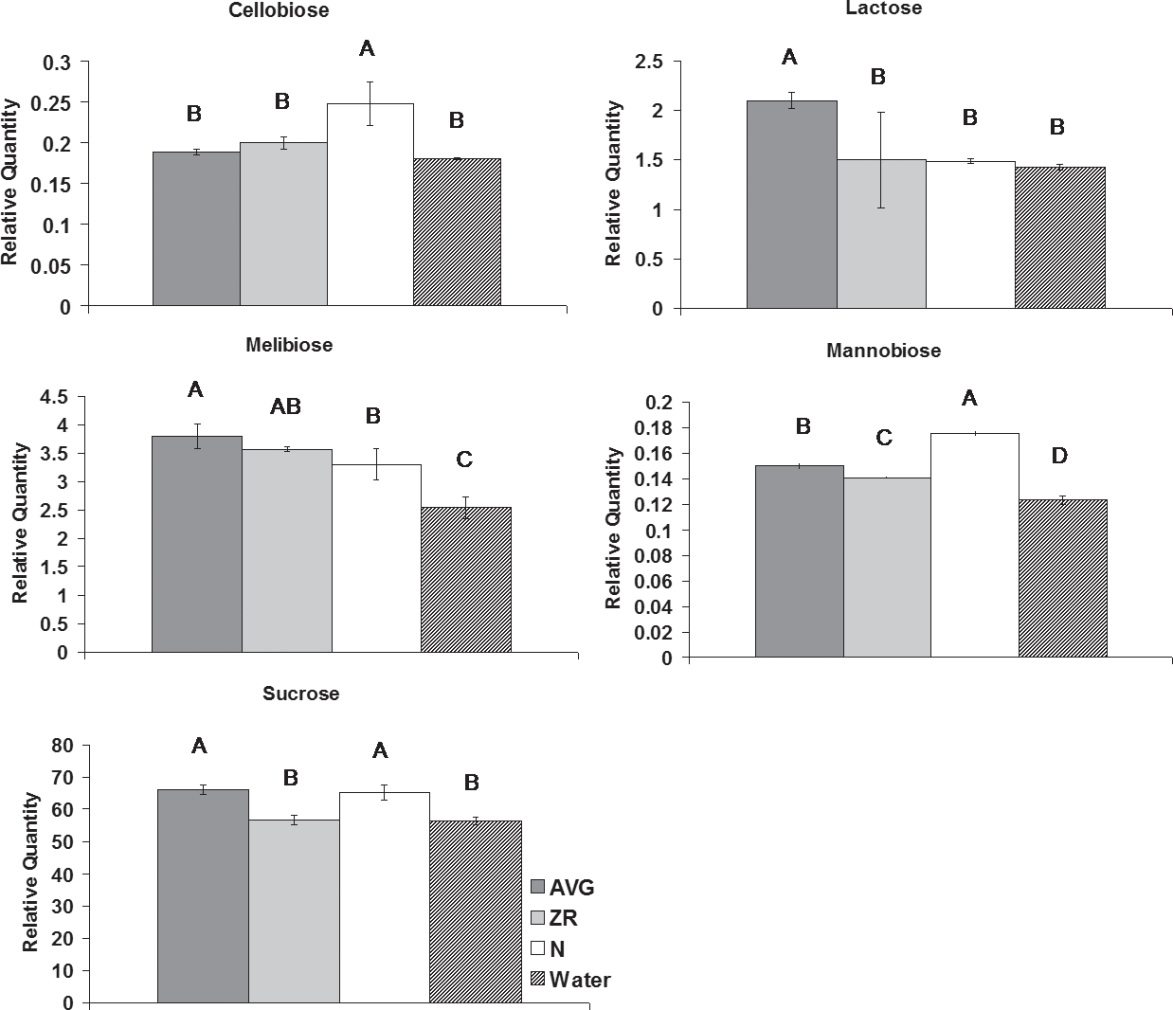
Disaccharide levels during heat stress for AVG, ZR, and N-treated plants, and the water-treated control. Relative quantities of disaccharides at 28-d heat stress for AVG, ZR, and N-treated plants, and the water-treated control. Error bars indicate standard deviations and letters are statistical groups according to Fisher’s protected LSD (p = 0.05), with groups not containing the same letter being significantly different. Only metabolites which had at least one group significantly different from the others are presented.

**Fig 5:**
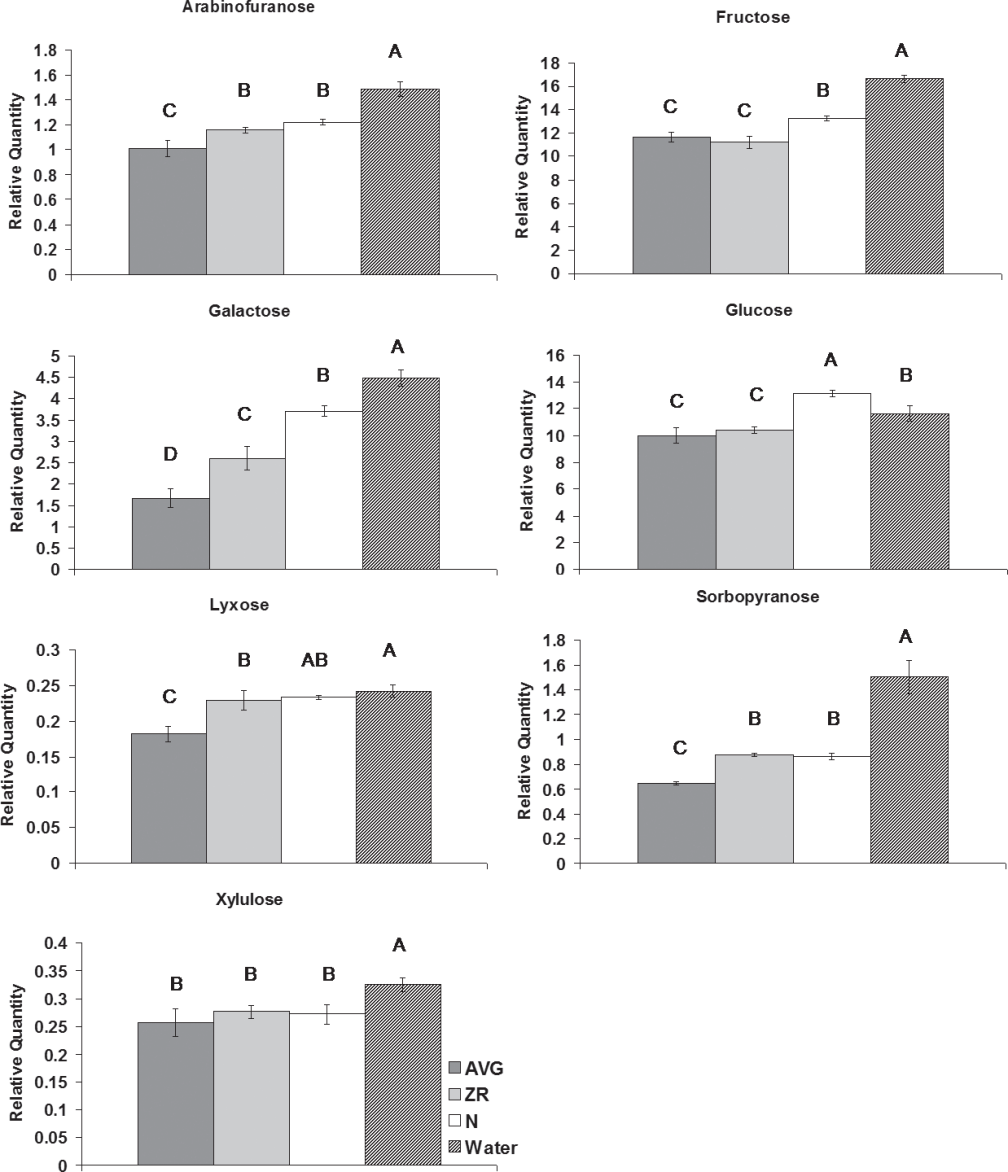
Monosaccharide levels during heat stress for AVG, ZR, and N-treated plants, and the water-treated control. Relative quantities of monosaccharides at 28-d heat stress for AVG, ZR, and N-treated plants, and the water-treated control. Error bars indicate standard deviations and letters are statistical groups according to Fisher’s protected LSD (p = 0.05), with groups not containing the same letter being significantly different. Only metabolites which had at least one group significantly different from the others are presented.

**Fig 6:**
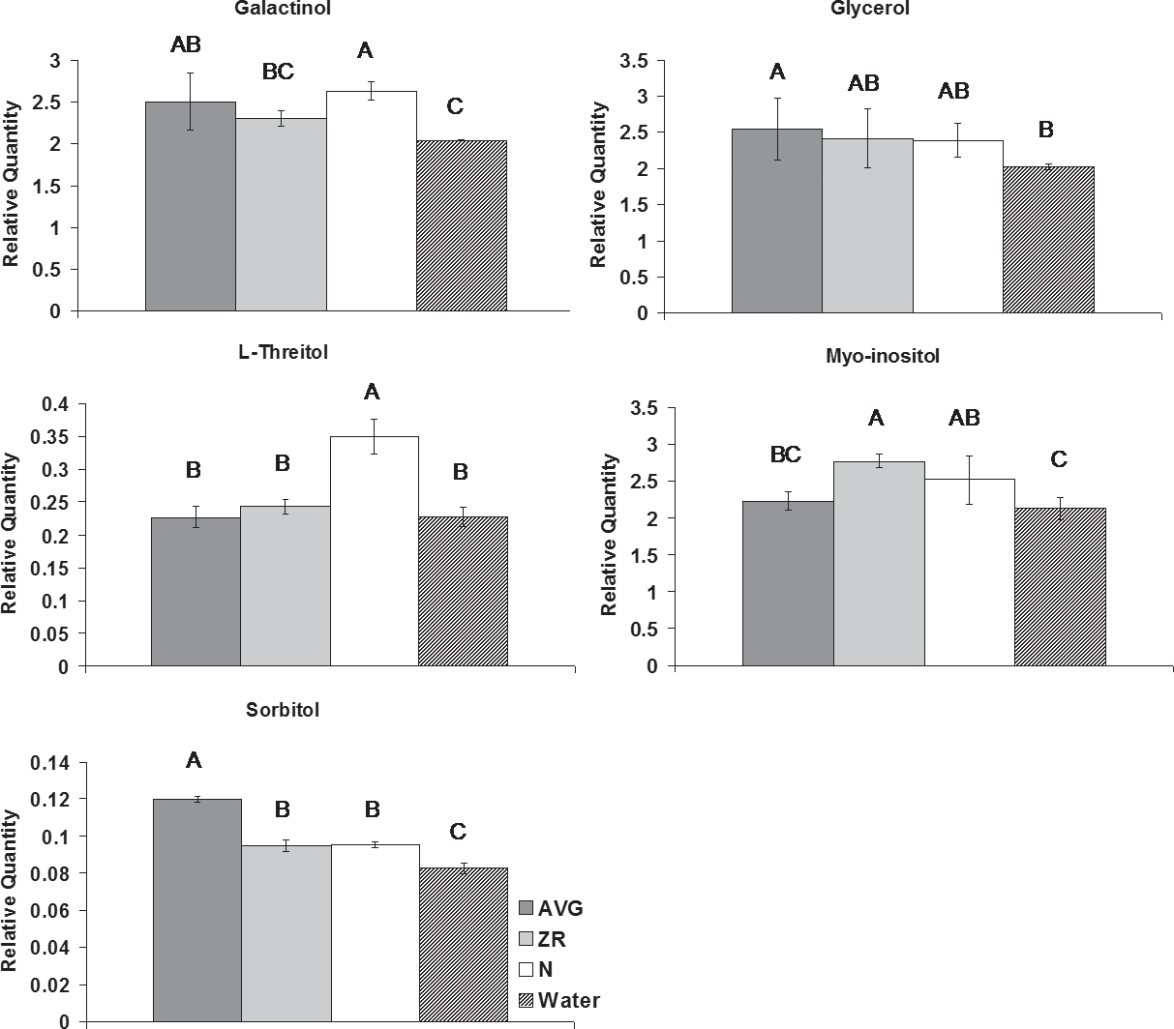
Sugar alcohol levels during heat stress for AVG, ZR, and N-treated plants, and the water-treated control. Relative quantities of sugar alcohols at 28-d heat stress for AVG, ZR, and N-treated plants, and the water-treated control. Error bars indicate standard deviations and letters are statistical groups according to Fisher’s protected LSD (p = 0.05), with groups not containing the same letter being significantly different. Only metabolites which had at least one group significantly different from the others are presented.

For sugars, application of N increased the level of cellobiose, glucose, and L-threitol compared to the untreated plants (Figs 4–6). Treatment with AVG resulted in the increase of lactose and glycerol content. Treatment with N or AVG also caused an increase in galactinol and sucrose. The application of ZR or N caused an increase in myo-inositol content. The application of ZR or AVG reduced the level of glucose, and lyxose. The content of glucose increased with N-treatment during heat stress compared to control plants. All three exogenous treatments caused an increase in mannobiose, melibiose, and sorbitol. Six sugars (arabinofuranose, fructose, galactose, maltose, sorbopyranose, and xylulose) exhibited decline in their content with N, AVG or ZR treatment.

**Fig 7:**
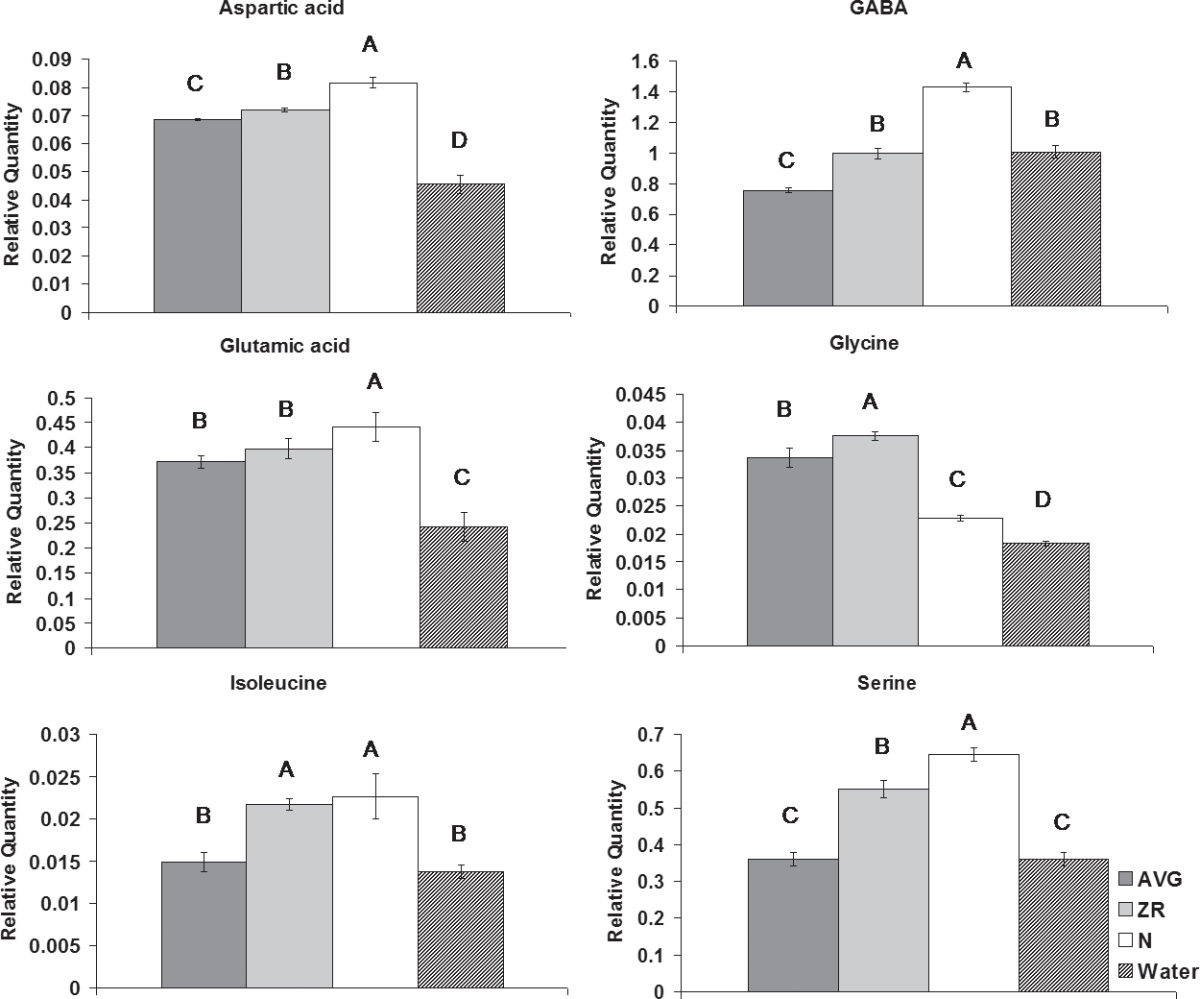
Amino acid levels during heat stress for AVG, ZR, and N-treated plants, and the water-treated control. Relative quantities of amino acids at 28-d heat stress for AVG, ZR, and N-treated plants, and the water-treated control. Error bars indicate standard deviations and letters are statistical groups according to Fisher’s protected LSD (p = 0.05), with groups not containing the same letter being significantly different. Only metabolites which had at least one group significantly different from the others are presented.

The application of N, ZR and AVG had differential effects on individual amino acids. Treatment with AVG resulted in decreases of threonine and gamma-aminobutyric acid (GABA), while N treatment caused an increase in the content of GABA (Fig 7). Both ZR and N treatment resulted in an increase in serine and isoleucine content. All three exogenous treatments resulted in increases in the content of aspartic acid, glutamic acid, and glycine.

### Classification of major metabolites responsive to N, ZR, or AVG and associated contribution to suppression of heat injury

**Fig 8:**
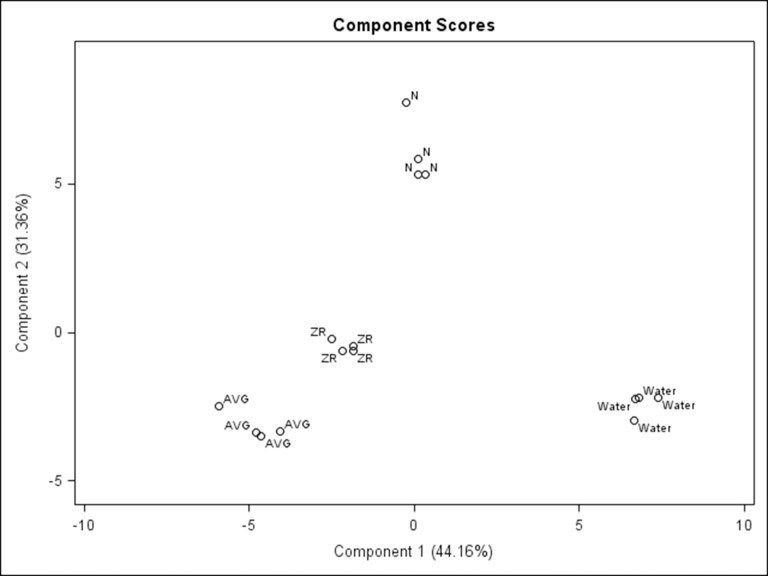
Principle component plot of AVG, ZR, N or water treatment groups during heat stress. Principle component analysis based on metabolite levels during 28 d heat stress for plants treated with AVG, ZR, N, or the water control. Component one is represented on the X-axis and accounted for 44.2% of variance, and component 2 is represented on the Y axis and accounted for 32.4% of variance.

Principle component analysis (PCA) separated metabolites responsive to N, ZR, or AVG under heat stress into different principle components, and determined the contribution of each component to the overall variations in metabolite accumulation due to the effects of N, ZR, or AVG (Fig 8). PCA was also used to identify metabolites which had the greatest contribution to the differences between treatment groups. Metabolites with the highest eigenvalues for a principle component (top 15%) were considered to have the greatest impact for a component. The first and second principle component accounted for 44.2% and 31.4% of variations in metabolite responses to N, ZR, or AVG treatments, respectively. Metabolite responses to untreated control were distinctly separated from those responsive to N, ZR, or AVG application within the first component of PCA while those responsive to N were distinctly separated into a group from those responsive to AVG or ZR treatment within the second component of PCA analysis.

**Fig 9:**
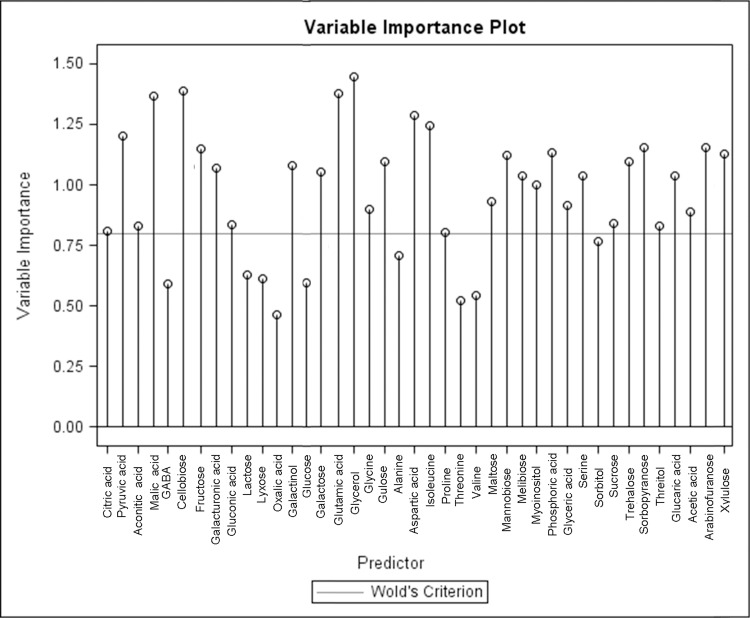
Variable importance plot of metabolites during heat stress. Variable importance projection plot generated from the partial least squares regression highlighting which metabolites may potentially play a larger role in heat tolerance as predicted by the model. Wold’s citerion is denoted by a horizontal line.

Partial least squares regression (PLS) was performed to confirm specific metabolites which may play a role in overall heat tolerance by modeling the relationship between metabolite levels and chlorophyll content as an indicator of heat-induced leaf senescence, subsequent variable importance projections were plotted (Fig 9). A number of metabolites, which are of potential importance for differences between treatments groups resulting in suppressed leaf senescence, were identified by both PCA and PLS analyses including sugars (arabinofuranose, fructose, galactose, melibiose, and sorbopyranose), sugar alcohol (sorbitol), and organic acids (citric acid). Other metabolites of potential importance were identified by one of the two analyses, including glycine (in PCA analysis) and sucrose (in PLS analysis).

## Discussion

Physiological results demonstrated that exogenous applications of AVG, ZR, or N resulted in the suppression of heat-induced leaf senescence or improvement in heat tolerance of creeping bentgrass, as manifested by increased TQ, chlorophyll content and membrane stability. The positive physiological effects of those compounds that could be associated with metabolic changes found through metabolic profiling are discussed below.

### Accumulation of sugars and sugar alcohols as affected by AVG, N, and ZR in relation to heat-induced leaf senescence

The most abundant disaccharide found in this study was sucrose. Sucrose is a major soluble sugar in plants for the storage and transport of carbon fixed through photosynthesis [34]. Higher levels of sucrose have been associated with enhanced heat tolerance in grass species [29, 35]. PLS analysis also associated sucrose levels with delayed leaf senescence as indicated by chlorophyll content levels in this study, indicating that it may be an important metabolite for stress tolerance. In addition to its primary role in metabolism, sucrose can also act as a signaling molecule effecting the regulation of other metabolic pathways [36]. Other disaccharide species, including lactose and melibiose accumulated during stress events can act as protective osmolytes which help maintain the integrity of membranes and proteins, as well as maintain cell hydration levels [37, 38]. The effects of AVG, ZR, and N on heat stress in creeping bentgrass were previously found to result in the up-regulation photosynthetic proteins such as ribulose-1,5-bisphosphate carboxylase oxygenase and chlorophyll a/b binding protein potentially resulting in an increase in carbohydrate synthesis [4]. Increased levels of cellobiose, lactose, mannobiose, melibiose, and sucrose found with the AVG and N treatment reflected active photosynthetic supply of carbohydrates and increased carbohydrate reserves as disaccharides, which could play roles in protecting leaves of creeping bentgrass from prolonged periods of heat stress.

Monosaccharides, such as glucose and fructose produced through photosynthesis are readily utilized in respiratory metabolism for energy production, which is critically important for plant survival of stresses [39]. With the exception of increases in the content of glucose with N treatment, the content of all other monosaccharides decreased with exogenous application of N, ZR, or AVG under heat stress. During heat stress respiration rate and respiratory demand for monosaccharides typically increase, which may lead to reduced accumulations of those sugars [1, 40]. Decreases in content of monosaccharides, including glucose and fructose, during heat stress have previously been reported in grass species [29, 35, 41]. The maintenance of respiration levels may represent more actively growing tissue, as well as generation of ATP for important stress defense mechanisms such as antioxidative functions, although increased amount of sugars may be consumed or simple sugar content may decline [42, 43]. Both PCA and PLS analysis associated fructose levels with differences in heat tolerance between treatment groups, supporting that it may be an important factor affecting heat tolerance. The decrease in the content of monosaccharides (arabinose, fructose, galactose, glucose, lyxose, sorbopyranose, and xylulose) in N, AVG or ZR-treated plants compared to the untreated control during heat stress may reflect an increase in carbohydrate consumption or utilization for the maintenance of respiratory metabolism under heat stress.

Sugar alcohols or polyols, such as galactinol, glycerol, myo-inositol, and sorbitol are reduced sugars containing several hydroxyl groups, which act as compatible solutes regulating osmotic adjustment and protecting cells from dehydration damages [44–47]. Sugar alcohols also have the ability to act as antioxidants which neutralize reactive oxygen species [48, 49]. Additionally myo-inositol has a role in signal transduction, as it binds to other molecules to form secondary messengers [50]. The greater accumulation of sugar alcohols in plants treated with AVG, ZR, or N compared to the untreated control under heat stress suggested that those sugar alcohols could be involved in the suppression of heat-induced leaf senescence by AVG, ZR, or N.

### Accumulation of organic acids as affected by AVG, N, and ZR in relation to heat-induced leaf senescence

Exogenous treatment with AVG, ZR or N resulted in higher accumulations of organic acids (aconitic acid, citric acid, galacturonic acid, glucaric acid, malic acid, oxalic acid, phosphorphoric acid, and pyruvic acid) compared to the untreated plants exposed to heat stress. Many of these organic acids with higher accumulation are intermediates of the tricarboxylic acid (TCA) cycle of respiration, including citric acid, aconitic acid, malic acid, and pyruvic acid. The increase in those TCA intermediates may represent more active mitochondrial respiration for the generation of ATP, but many of these intermediates can also feed into other metabolic pathways, which are important for regulating various cellular functions, such as nitrogen assimilation and redox balance [51]. The increased accumulation of organic acids in AVG, ZR, or N-treated plants corresponded with the decreased content of monosaccharides as substrates in respiration, which together suggested that the application of N, AVG, and ZR could help leaves of creeping bentgrass maintain more active energy metabolism and also reflected the positive effects of those treatments on the alleviation of leaf senescence or damages induced by heat stress.

### Accumulation of amino acids as affected by AVG, N, and ZR in relation to heat-induced leaf senescence

The application of N, ZR, and AVG had differential effects on different amino acids content. The application of N resulted in increases in the content of GABA, glycine, aspartic acids, glutamic acid, and serine. Amino acids are major nitrogen containing cellular constituents [19]. The increase in amino acid content with exogenous applications of N may be due to higher levels of nitrogen available for the plant to assimilate into amino acids. Nitrogen status has previously been shown to affect free amino acid content [52, 53]. GABA is a non-protogenic amino acid which has also been found to increase in response to stresses [54] potentially serving as a signaling molecular, and also participates in other metabolic processes, including carbon and nitrogen balance [55, 56]. The increased accumulation of GABA with N treatment under heat stress suggested that this metabolite could play protective roles of leaves from heat-induced leaf senescence, although the underlying mechanisms deserve further investigation.

Higher accumulations of aspartic acid, glutamic acid, and glycine were observed with AVG, ZR, and N treatment under heat stress. Aspartic acid is a major precursor to many other amino acids [57] including isolucience and glycine and its accumulation may represent an important shift in amino acid metabolism for enhanced heat tolerance. Similar to aspartic acid, glutamic acid is another amino acid which is a precursor to many other amino acids [58]. Additionally glutamic acid plays important roles in nitrogen metabolism as well as chlorophyll biosynthesis [59]. The increase in glutamic acid may improve heat tolerance by improving chlorophyll production, as well as improving the integration of nitrogen into other cellular molecules. Glycine is a product of photorespiration and is the precursor for the synthesis of glutathione, purines, and porphyrins [60, 61]. PCA analysis found glycine to be one of the metabolites responsible for a greater percent of variance between treatment groups. Increased accumulations of glycine may represent a more active photorespiration cycle averting heat stress damages.

Both ZR and N treatments also enhanced the accumulation of isoleucine and serine in plants exposed to heat stress. Serine is another important amino acid involved the recycling of metabolites during the photorespiratory cycle which has been associated with improved stress tolerance [62]. Isoleucine can feed into the TCA cycle, as well as help generate redox potential, to help maintain respiration rates [63], and has also shown to play roles in jasmonate signaling [64]. The increase in isoleucine may potential increase heat tolerance by helping maintain signaling pathways or TCA cycle activity. Proteomic profiling of plants treated with AVG ZR or N during heat stress found increases in proteins, such as glycine decarboxylase or aminomethyltransferase associated with photorespiratory glycine cleavage, or ferredoxin dependent glutamate synthase support shifts in metabolism associated with these amino acids [3]. Metabolite results indicated that the enhanced accumulation of those amino acids associated with nitrogen balance, photorespiration or which are important biosynthetic precursors may represent important shift in metabolism resulting in delayed heat-induced senescence.

## Conclusions

Exogenous treatment of creeping bentgrass with AVG, ZR or N resulted in improved heat tolerance compared to an untreated control, demonstrated by improved membrane stability, chlorophyll content and overall quality. In general metabolic changes included increased amino acid, organic acid, disaccharide and sugar alcohol content, and lower monosaccharide content with exogenous treatment with AVG, ZR or N compared to the untreated control under heat stress, which are involved in osmoregulation, antioxidant metabolism, carbon and nitrogen metabolism, as well as stress signaling molecules. Future research will identify underlying mechanism how changes in individual metabolites affected by AVG, N, or ZR may be involved in the suppression of heat-induced leaf senescence in cool-season grass species. Such information will provide further insights into metabolic and molecular factors controlling heat-induced leaf senescence and also is useful to identifying metabolites that may be incorporated into chemical products alleviating heat-induced leaf senescence through exogenous applications in cool-season turfgrass management.

## Supporting Information

S1 Table Metabolite levels during heat stress for AVG, ZR, N-treated and control plants. Relative quantities of metabolites and statistical groupings for AVG, ZR, N and Control treatments at 28 days heat stress. Relative values are calculated from an internal ribitol standard. Letters represent LSD groupings for a given metabolite with treatments sharing a letter not being significantly different at p = 0.05.
(DOC)
