# Supplementary material for: Metabolite Responses to Exogenous Application of Nitrogen, Cytokinin, and Ethylene Inhibitors in Relation to Heat-Induced Senescence in Creeping Bentgrass
Source: PLoS One. 2015 Mar 30;10(3):e0123744. doi: 10.1371/journal.pone.0123744 (PMC4379107; doi:10.1371/journal.pone.0123744)
Supplement: S1 Table — Relative quantities of metabolites and statistical groupings for AVG, ZR, N and Control treatments at 28 days heat stress. Relative values are calculated from an internal ribitol standard. Letters represent LSD groupings for a given metabolite with treatments sharing a letter not being significantly different at p = 0.05. (DOC) [file pone.0123744.s001.doc]

Table S1: Relative values of metabolites and statistical groupings for each treatment under 28 days heat stress conditions.

|  | **AVG** | | **ZR** | | **N** | | **Control** | | **LSD value** |
| --- | --- | --- | --- | --- | --- | --- | --- | --- | --- |
| **Metabolite:** | Relative value | LSD group | Relative value | LSD group | Relative value | LSD group | Relative value | LSD group |
| Acetic acid | 0.215 | A | 0.232 | A | 0.129 | B | 0.234 | A | 0.025 |
| Aconitic acid | 3.699 | A | 2.658 | B | 2.129 | C | 1.781 | D | 0.275 |
| Citric acid | 1.316 | A | 1.219 | B | 1.134 | C | 0.889 | D | 0.063 |
| Galacturonic acid | 0.165 | C | 0.218 | B | 0.242 | A | 0.171 | C | 0.015 |
| Glucaric acid | 0.368 | C | 0.436 | B | 0.544 | A | 0.368 | C | 0.037 |
| Gluconic acid | 0.176 | A | 0.163 | A | 0.056 | A | 0.164 | A | 0.356 |
| Glyceric Acid | 0.670 | A | 0.869 | A | 2.162 | A | 0.629 | A | 2.385 |
| Malic acid | 2.674 | A | 2.754 | A | 3.063 | A | 1.820 | B | 0.472 |
| Oxalic acid | 2.703 | A | 2.338 | B | 2.024 | C | 2.122 | C | 0.187 |
| Phosphoric acid | 0.021 | AB | 0.025 | AB | 0.026 | A | 0.015 | B | 0.011 |
| Pyruvic acid | 0.015 | A | 0.006 | BC | 0.008 | B | 0.004 | C | 0.004 |
| Arabinofuranose | 1.008 | C | 1.157 | B | 1.217 | B | 1.484 | A | 0.074 |
| Cellobiose | 0.189 | B | 0.199 | B | 0.248 | A | 0.181 | B | 0.020 |
| Fructose | 11.667 | C | 11.228 | C | 13.231 | B | 16.624 | A | 0.668 |
| Galactinol | 2.506 | AB | 2.304 | BC | 2.634 | A | 2.038 | C | 0.289 |
| Galactose | 1.678 | D | 2.604 | C | 3.721 | B | 4.484 | A | 0.330 |
| Glucose | 10.009 | C | 10.438 | C | 13.104 | A | 11.634 | B | 0.778 |
| Glycerol | 2.538 | A | 2.411 | AB | 2.385 | AB | 2.021 | B | 0.486 |
| Gulose | 9.778 | A | 8.773 | A | 10.504 | A | 8.274 | A | 2.238 |
| Lactose | 2.097 | A | 1.501 | B | 1.486 | B | 1.424 | B | 0.399 |
| L-Threitol | 0.227 | B | 0.243 | B | 0.350 | A | 0.227 | B | 0.030 |
| Lyxose | 0.182 | C | 0.229 | B | 0.233 | AB | 0.242 | A | 0.012 |
| Maltose | 0.743 | C | 0.676 | D | 0.937 | B | 1.018 | A | 0.033 |
| Mannobiose | 0.150 | B | 0.141 | C | 0.176 | A | 0.124 | D | 0.004 |
| Melibiose | 3.808 | A | 3.580 | AB | 3.306 | B | 2.540 | C | 0.285 |
| Myo-inositol | 2.232 | BC | 2.772 | A | 2.523 | AB | 2.128 | C | 0.314 |
| Sorbitol | 0.120 | A | 0.095 | B | 0.095 | B | 0.083 | C | 0.004 |
| Sorbopyranose | 0.646 | C | 0.876 | B | 0.860 | B | 1.505 | A | 0.106 |
| Sucrose | 66.112 | A | 56.702 | B | 65.171 | A | 56.317 | B | 3.079 |
| Trehalose | 0.196 | A | 0.131 | C | 0.163 | B | 0.203 | A | 0.011 |
| Xylulose | 0.257 | B | 0.276 | B | 0.272 | B | 0.325 | A | 0.031 |
| Alanine | 0.339 | B | 0.363 | B | 0.440 | A | 0.464 | A | 0.026 |
| Aspartic acid | 0.069 | C | 0.072 | B | 0.082 | A | 0.046 | D | 0.003 |
| GABA | 0.755 | C | 0.998 | B | 1.433 | A | 1.008 | B | 0.046 |
| Glutamic acid | 0.371 | B | 0.398 | B | 0.441 | A | 0.242 | C | 0.036 |
| Glycine | 0.034 | B | 0.038 | A | 0.023 | C | 0.018 | D | 0.002 |
| Isoleucine | 0.015 | B | 0.022 | A | 0.023 | A | 0.014 | B | 0.002 |
| Proline | 0.428 | B | 0.316 | C | 0.649 | A | 0.661 | A | 0.029 |
| Serine | 0.359 | C | 0.549 | B | 0.644 | A | 0.359 | C | 0.034 |
| Threonine | 0.254 | C | 0.308 | BC | 0.442 | A | 0.361 | B | 0.057 |
| Valine | 0.144 | D | 0.152 | C | 0.195 | A | 0.165 | B | 0.004 |
